# Increased injury severity and age-related mortality in trauma victims after e-bike versus conventional bicycle accidents: a concerning call to action!

**DOI:** 10.1186/s13037-026-00494-y

**Published:** 2026-06-20

**Authors:** Deniz D. Özman, Sabine M. I. Roth, Maria Gabriella Fois, Luca Diehr, Michel P. J. Teuben, Huanglin Zhang, Max Praster, Till Berk, Frank Hildebrand, Rald V. M. Groven

**Affiliations:** 1 Department of Orthopaedic, Trauma and Reconstructive Surgery, RWTH Aachen University Hospital, Pauwelsstreet 30, 52074 Aachen, Germany; 2 Department of Biohybrid & Medical Textiles (BioTex), AME-Institute of Applied Medical Engineering, Helmholtz Institute, RWTH Aachen University, Forckenbeckstreet 55, 52074 Aachen, Germany; 3 Department of Traumatology, University Hospital Zurich, Raemistreet 100, Zurich, 8091 Switzerland

**Keywords:** e-Bike accidents, Geriatric trauma, Age-adjusted injury patterns, Polytrauma, Alcohol-related cycling accidents

## Abstract

**Background:**

Although e-bike accidents are of growing clinical relevance, there is limited large-scale research comparing injury patterns and outcomes between e-bike (EB) and conventional bicycle (CB) accidents in large patient cohorts. This study performed comparative analyses of both groups to identify patients at risk and guide future prevention and clinical management strategies.

**Methods:**

A retrospective analysis of the TraumaRegister DGU^®^ was conducted. Patients aged 16 years or older who sustained severe injuries (AIS ≥ 3 in minimally one body region) in accidents involving conventional bicycles or e-bikes between January 2020 and December 2023 were included. Patient demographics, injury patterns, trauma severity, treatment characteristics, and clinical outcomes were analyzed.

**Results:**

A total of 9,170 bicycle accident cases were included (EB *n* = 1,160; CB *n* = 8,010). EB riders were significantly older than CB riders (median age 63 years; IQR 53-73 vs. 57 years IQR 44-69; *p* < 0.001) and more frequently sustained polytrauma (16.7% vs. 12.3%; *p* < 0.001). Compared with CB riders, EB riders more often suffered injuries to the head (67.2% vs. 56.2%; *p* < 0.01), face (22.7% vs. 17.8%; *p* < 0.001), and chest (55.2% vs. 51.8%; *p* = 0.030), and were more likely to sustain injuries affecting multiple body regions (*p* < 0.001). Primary ICU treatment was required more frequently after EB accidents (70.3% vs. 63.5%; *p* < 0.001). Age-stratified analyses showed that younger EB riders were more frequently involved in nighttime and alcohol-related accidents, whereas mortality increased significantly with age, from 2.7% in patients aged 16-59 years to 18.6% in those aged ≥ 80 years.

**Conclusions:**

E-bike accidents are associated with a higher prevalence of head, face, and chest injuries, increased rates of polytrauma and multi-region injuries, and a greater need for ICU treatment compared with conventional bicycle accidents. These differences are particularly relevant in older riders, who represent the majority of severely injured e-bike users and experience substantially higher mortality rates. Targeted prevention strategies, improved protective measures, and age-specific risk communication may help reduce the burden of e-bike-related injuries.

## Background

Urban mobility patterns in Europe have undergone remarkable changes in recent years, largely driven by the significant increase in the use of electric bicycles (e-bikes) [1, 2]. This growing popularity can be attributed to several factors, including their efficiency, extended ranges, and the reduced physical effort required for use. These characteristics have made e-bikes particularly appealing to older adults, individuals with overall lower physical fitness levels, and, more recently, to a growing segment of young consumers [2, 3].

Against this background, cycling-related accidents have become more prevalent and represent a significant public health issue, in particular in the western world [4]. Such accidents can result in severe injuries, including traumatic brain injury, bone fractures, blunt trauma to the chest and/or abdomen, and diffuse soft-tissue damage [5, 6]. E-bikes are bicycles equipped with an electric motor that provides pedal-assisted propulsion. In Europe, most e-bikes provide motor assistance up to 25 km/h with a maximum continuous motor output of 250 W, although faster variants are also available. Compared with conventional bicycles, e-bikes are generally heavier due to the integrated battery and motor system and have become increasingly popular because they enable longer travel distances with reduced physical effort. The emergence of e-bikes introduced additional concerns as compared to regular bicycles due to higher travelling speeds and greater overall weight resulting from integrated motor and battery systems [4]. These two factors, velocity and mass, increase the cyclists’ kinetic energy, resulting in substantially higher impact forces during accidents [7].

Although research on trauma mechanisms and demographics on regular cycling accidents exists, literature focusing on injury patterns and the trauma severity of injuries sustained in accidents involving e-bikes versus conventional bicycles in large patient cohorts is lacking [4]. Therefore, the current study aimed to compare injury patterns, injury severity, and clinical outcomes between patients involved in e-bike and conventional bicycle accidents using data from the TraumaRegister DGU^®^. We hypothesized that patients involved in e-bike accidents would sustain more severe injuries, exhibit a higher prevalence of polytrauma and injuries affecting multiple body regions, and require more intensive hospital treatment than patients involved in conventional bicycle accidents.

## Methods

The TraumaRegister DGU^®^ of the German Trauma Society (Deutsche Gesellschaft für Unfallchirurgie, DGU) was founded in 1993. The aim of this multi-centre database is a pseudonymised and standardised documentation of severely injured patients.

Data are collected prospectively in four consecutive time phases from the site of the accident until discharge from hospital:

A. Pre-hospital phase,
B. Emergency room and initial surgery,
C. Intensive care unit and.
D. Discharge.

The documentation includes detailed information on demographics, injury pattern, comorbidities, pre- and in-hospital management, course on intensive care unit, relevant laboratory findings including data on transfusion and outcome of each individual. The inclusion criterion is the admission to hospital via the emergency room with subsequent Intesive Care Unit treatment or reaching the hospital with vital signs but death before admission to the ICU. The infrastructure for documentation, data management, and data analysisis provided by AUC - Academy for Trauma Surgery (AUC - Akademie der Unfallchirurgie GmbH), a company affiliated to the German Trauma Society. The scientific leadership is provided by the Committee on Emergency Medicine, Intensive Care and Trauma Management (Sektion NIS) of the German Trauma Society. The participating hospitals submit their data pseudonymised into a central database via a web-based application. Scientific data analysis is approved according to a peer review procedure laid down in the publication guideline of TraumaRegister DGU^®^.

The participating hospitals are primarily located in Germany (90%), but a rising number of hospitals of other countries contribute data as well (at the moment from Austria, Belgium, China, Finland, Luxembourg, Slovenia, Switzerland, The Netherlands, and the United Arab Emirates). Currently, more than 30,000 cases from around 700 hospitals are entered into the database per year [8, 9]. Participation in TraumaRegister DGU^®^ is voluntary. For hospitals associated with TraumaNetzwerk DGU^®^, however, the entry of at least a basic data set is obligatory for reasons of quality assurance.

### Inclusion & exclusion criteria

For this study, data were used from patients within the DACH-Region (Germany, Austria, and Switzerland) who suffered from an accident while riding either a conventional bike (CB) or an e-bike (EB) between the 1st of January 2020 and the 31st of December 2023 who were included in the TR-DGU. For age-stratified analyses, all patients with an Abbreviated Injury Scale (AIS) score of ≥ 3 in at least one body region were included. To analyze injury patterns, all injuries with an AIS Score of ≥ 2 were included to include all non-minor injuries as well. Patients were eligible for inclusion if they were ≥ 16 years of age. The following four age groups were defined; 16–59, 60–69, 70–79, and 80 + years of age. This stratification was performed because injury mechanisms and clinical outcomes are comparable below 60 years of age, whereas age-related differences become increasingly evident and clinically relevant in older patients. Patients were excluded if they were transferred out to another hospital in the early (< 48 h) post-traumatic phase, in order to avoid double counting.

### Definitions & outcome measures

The Abbreviated Injury Scale (AIS, Version 2005/Update 2008), developed by the Association for the Advancement of Automotive Medicine (AAAM), was used to code injuries and classify their severity. Each injury is categorized into nine predefined anatomical regions and graded by severity on a scale from 1 (minor injury) to ≥ 5 (critical, life-threatening up to fatal injury). Weekend admissions were defined as admissions from Friday to Sunday. Nighttime admissions were defined from 06:00 p.m. until 06:00 a.m. Polytrauma was defined according to the Berlin consensus definition by Pape et al. [10]. Age ≥ 65 years was defined as geriatric trauma.

The primary outcome of this study was age-adjusted in-hospital mortality following e-bike and conventional bicycle accidents. Secondary outcomes included the prevalence of injuries affecting multiple body regions (AIS ≥ 2), injury distribution according to body region, Injury Severity Score (ISS), prevalence of polytrauma, admission to the intensive care unit (ICU), operative treatment, hospital and ICU length of stay, and age-stratified injury characteristics.

### Statistical analyses & ethical approval

For this study, the complete TR-DGU data set was retrospectively analyzed in anonymized form using SPSS (Version 29, IBM Inc., Armonk, NY, USA). The corresponding data were provided alongside case numbers and associated percentages, as well as mean values and standard deviations. The level of significance was set to alpha < 0.05. Patients were included in the registry upon informed consent. Database inclusion, analyses, and interpretation of the results were all performed in accordance with the Declaration of Helsinki in its most recent form, the European General Data Protection Regulation, and the Federal Data Protection Act Germany. No a priori sample size calculation was performed, as all eligible patients recorded in the TraumaRegister DGU^®^ during the predefined study period were included in the analysis. The present manuscript was written with adherence to the publication guidelines of the TR-DGU and is registered and approved by the DGU review board under study number 2024-033.

## Results

### Patient demographics and patient related factors

**Fig. 1:**
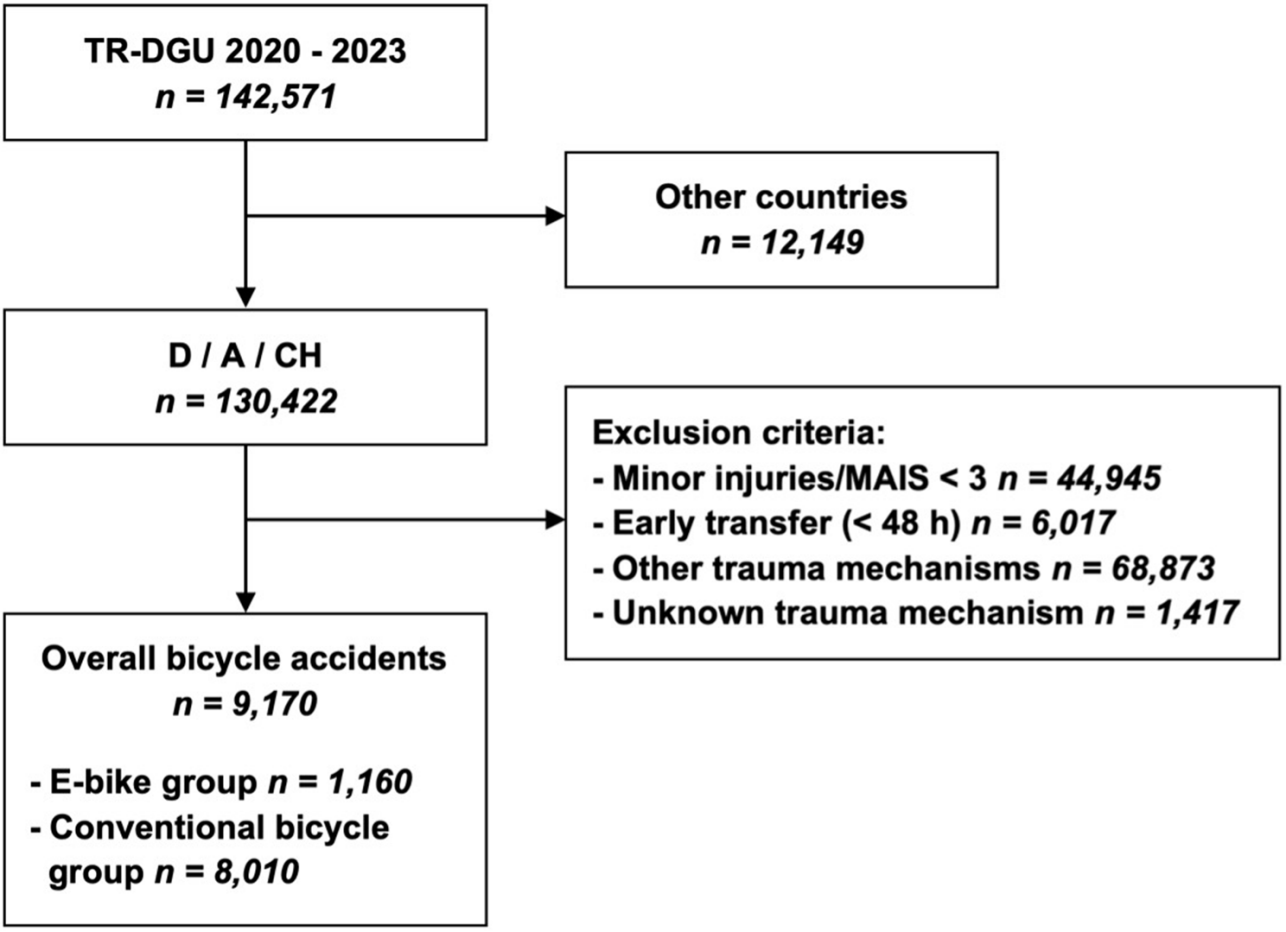
Flowchart depicting the patient cohort obtained from the TraumaRegister DGU^®^. Patients from Germany (D), Austria (A), and Switzerland (CH) were included in the study. Furthermore, patients with minor injuries/maximum AIS smaller than 3 were excluded, as well as patients who were transferred to another hospital within 48 h, or those with different or unknown trauma mechanisms

From the commencement of data collection on e-bike accidents in 2020 until December 31, 2023, a total of 9,170 bike accidents (CB *n* = 8,010; EB *n* = 1,160) from 601 hospitals were registered in the TR-DGU database (Fig. 1). Approximately two thirds of patients in the conventional bicycle (CB) as well as e-bike (EB) groups were men. Furthermore, patients in the EB group were significantly older than those in the CB group (*p* < 0.001) and were transported to the hospital by air ambulance significantly more often (*p* < 0.001) (all Table 1). The latter observation is consistent with the increased prevalence of polytrauma in the EB group (*p* < 0.001) (Table 3). During the study period, the incidence of accidents in the EB group increased more profoundly compared to the CB group. Seasonal trends were also observed, with accidents in both groups occurring significantly more in the summer (*p* = 0.035). No significant differences between the CB and EB groups were observed concerning the number of weekend or night admissions (all Table 1).

**Table 1:** Patient demographics and accident-related factors. Data are depicted as number and percentage, mean with standard deviation (SD), or median and interquartile range (IQR) as appropriate

	Conventional bicycle (*n* = 8010)	E-bike (*n* = 1160)	*p-value>*
**Female sex n (%)**	2076 (26.0%)	347 (30.0%)	0.004
**Age years (IQR)**	57 (44–69)	63 (53–73)	< 0.001
**On scene time (min)**	25 (18–34)	26 (18–35)	0.21
**Total time (min)**	58 (45–74)	60 (47–77)	< 0.001
**Injury Severity Score (IQR)**	17 (13–25)	18 (14–25)	<0.001
**Mode of transportation n (%)**			
EMS Emergency physician w/o emergency physician HEMS Privat transport	5521 (76.0%) *4307 (59.3%)* *1214 (16.7%)* 1562 (21.5%) 184 (2.5%)	748 (70.3%) *569 (53.5%)* *179 (16.8%)* 295 (27.8%) 20 (1.9%)	< 0.001
**Admissions per year n (%)**			
2020 2021 2022 2023	1836 (89.6%) 1927 (88.3%) 2153 (86.1%) 2094 (85.9%)	214 (10.4%) 256 (11.7%) 347 (13.9%) 343 (14.1%)	-
**Season n (%)**			
Winter Spring Summer Autumn	718 (9.0%) 2080 (26.0%) 3346 (41.8%) 1866 (23.2%)	83 (7.2%) 278 (24.0%) 527 (45.4%) 272 (23.4%)	0.035
**Weekend admission n (%)**			
Yes	3620 (45.2%)	530 (45.7%)	0.75
**Time of admission n (%)**			
Night (6 PM – 6 AM)	2808 (35.1%)	421 (36.3%)	0.41

EMS: ground-based emergency medical service, HEMS: helicopter emergency medical service

### Injury patterns

**Fig. 2:**
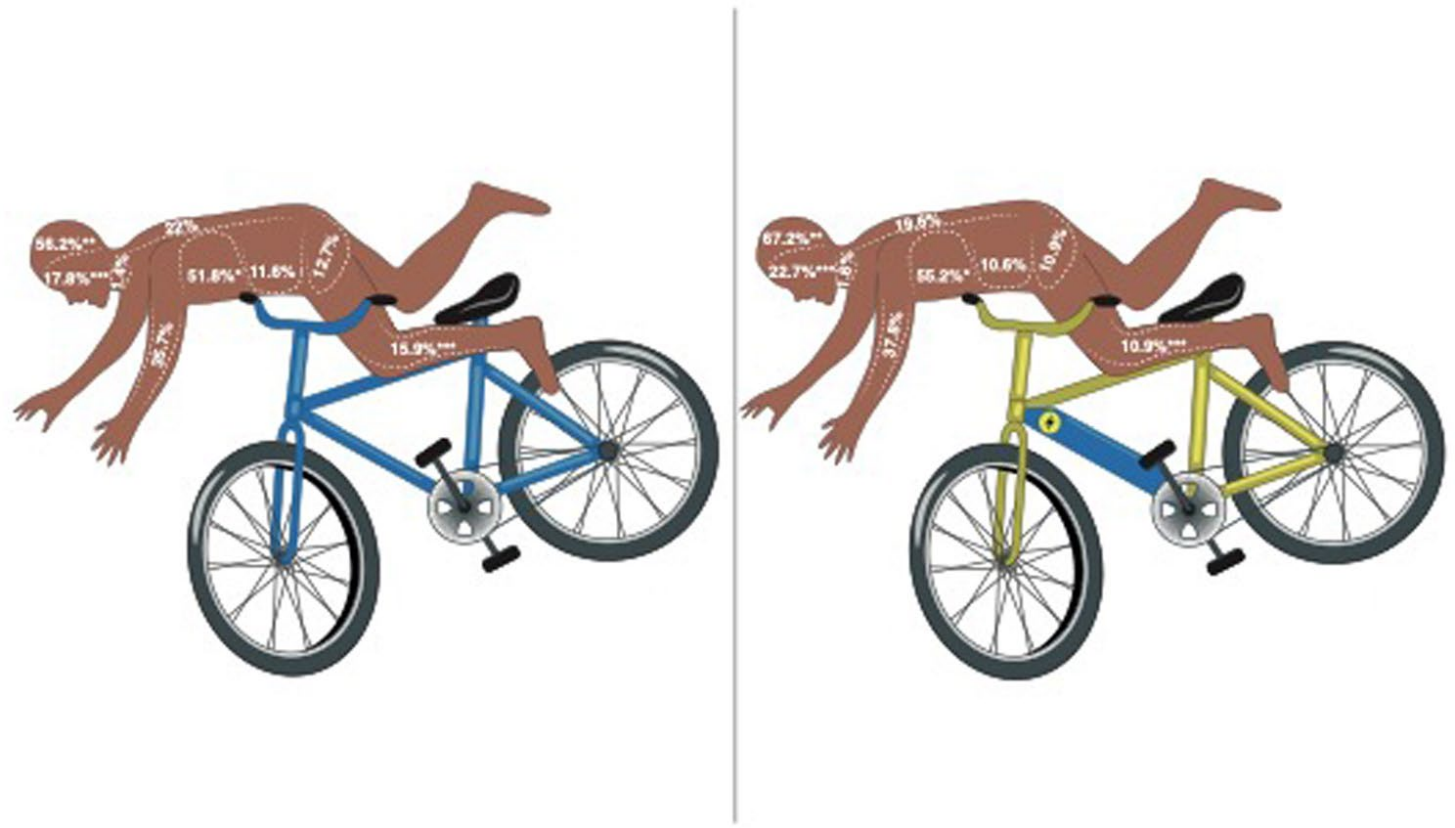
Injury distribution in the CB (left; *n* = 8,010) and EB (right; *n* = 1,160) groups. Data are represented as percentages. Significant differences between the CB and EB groups are marked in the respective body regions as follows: * *p* < 0.05, ** *p* < 0.01, *** *p* < 0.001

Cyclists in the EB group sustained significantly more head, face, and chest injuries than those in the CB group (all *p* < 0.05, Fig. 2; Table 2), whereas injuries to the legs were significantly more prevalent in the CB group (*p* <0.001, Fig. 2). No significant differences in AIS scores for other body regions were identified between the two groups (Fig. 2). Finally, cyclists in the EB group sustained significantly more injuries with an AIS ≥ 2 to multiple body regions than those in the CB group (*p* < 0.001) (Table 2).

**Table 2:** Rate of injury to multiple body regions with an AIS (Abbreviated Injury Scale) ≥ 2, according to the 10 AIS regions

	Conventional bicycle (*n = 8010*)	E-bike (*n = 1160*)	*p*-value
Head	4501	779	< 0.01
Face	1428	263	< 0.001
Neck	116	19	0.62
Chest	4146	640	0.030
Abdomen	933	123	0.29
Spine	1763	227	0.059
Arms	2856	439	0.146
Legs	1272	126	0.001
Pelvis	1017	127	0.092
Number of body			
regions with AIS ≥ 2 n (%)			
1 2 3 4 ≥ 5	2435 (30.4%) 2697 (33.7%) 1803 (22.5%) 711 (8.9%) 364 (4.5%)	310 (26.7%) 367 (31.6%) 296 (25.5%) 139 (12.0%) 48 (4.2%)	< 0.001

### Trauma bay admission and early hospital phase

A significantly higher proportion of patients in the EB group were aged 65 years and over (*p* < 0.001) (Table 3). Furthermore, cyclists in the EB group were significantly more likely to sustain a polytrauma (*p* < 0.001). Significantly more accidents occurred while under the influence of alcohol in the EB group as compared to the CB group, 37.5% vs. 29.1% respectively. Also, accidents under the influence of alcohol occurred significantly more often at night (*p* < 0.001) (Table 3). Significantly more patients sustaining e-bike accidents required ICU treatment, while CB-patients were more likely to receive direct operative treatment after trauma bay admission (*p* < 0.001) (Table 3). No significant differences were observed between the groups in terms of ventilator days, nor overall hospital mortality.

**Table 3:** Admission criteria and trauma bay treatment characteristics. Data are depicted as number and percentage, mean with standard deviation, or median and interquartile range as appropriate

	Conventional bicycle (*n = 8010*)	E-bike (*n = 1160*)	*p*-value
**Polytrauma>***n* (%)	982 (12.3%)	194 (16.7%)	< 0.001
**WBCT n (%)**	5824 (73.5%)	879 (76.2%)	0.049
**Transfusion in trauma bay n (%)**	405 (5.1%)	51 (4.4%)	0.33
**Age ≥ 65 years n (%)**	2689 (33.6%)	526 (45.3%)	< 0.001
**Alcohol n (%)** if admission at daytime if admission at night	800 (29.1%) *283 (35.5%)* *517 (64.6%)*	154 (37.5%) *45 (29.3%)* *109 (70.7%)*	< 0.001
**Destination after trauma bay n (%)**			
Surgery Intensive Care Unit Other	1512 (18.9%) 5087 (63.5%) 1026 (17.6%)	164 (14.1%) 816 (70.3%) 147 (15.6%)	< 0.001
**Operative treatment n (%)**	5144 (64.2%)	706 (60.9%)	0.026
**Length of stay in days (± IQR)**			
Overall LOS in hospital Intensive Care Unit LOS	9 (5–16) 2 (1–5)	10 (6–16) 2 (1–5)	0.23 0.036
**Ventilated patients (%)**	653 (11.8%)	347 (13.3%)	0.54
**In hospital mortality n (%)**	626 (7.8%)	84 (7.2%)	0.49

WBCT; Whole-Body Computed Tomography, LOS; Length Of Stay, Other: normal ward, mortality in trauma bay

### Age stratified patient characteristics in the EB group

The sex distribution among all accidents in the EB group was not influenced by patient age. Younger patients in the EB group were significantly more likely to be involved in alcohol-related accidents and to be admitted to hospital at night than older patients (both *p* < 0.001) (Table 4). Traumatic brain injury occurred significantly more often in older patients and showed a marked increase from the age of 70 years onwards (*p* = 0.004). A significant sevenfold increase in mortality was observed among patients in the youngest age category and those aged 80 and above (*p* < 0.001) (Table 4).

**Table 4:** Specific clinical characteristics per age group for E-bike accidents AIS; Abbreviated Injury Scale

	16–59 years (*n = 482*)	60–69 years (*n = 298*)	70–79 years (*n = 249*)	80 + years (*n = 129*)	*p*-value
Female sex *n* (%)	151 (31.3%)	100 (33.6%)	65 (26.1%)	31 (24.0%)	0.10
Traumatic brain injury (AIS ≥ 2) n (%)	302 (62.7%)	195 (65.4%)	186 (74.7%)	94 (72.9%)	0.004
Operative treatment n (%)	291 (60.4%)	190 (63.8%)	148 (59.4%)	75 (58.1%)	0.63
In hospital mortality n (%)	13 (2.7%)	14 (4.7%)	33 (13.3%)	24 (18.6%)	< 0.001
Alcohol n (%)	106 (52.0%)	28 (30.1%)	13 (16.3%)	7 (21.2%)	< 0.001
Admission at night n (%)	235 (48.8%)	90 (30.2%)	72 (28.9%)	23 (17.8%)	< 0.001

## Discussion

In recent years, e-bikes have gained popularity and e-bike related accidents have subsequently become more prevalent [1]. They offer convenience, but everyday clinical practice has demonstrated that their use can be associated with severe injuries [4]. The present study is the first to determine patient characteristics, injury patterns and severity, as well as patient outcomes from conventional bicycle- and e-bike accidents in a multi-center setting. The main findings can be summarized as follows:

1. Patients in the EB group were significantly older, sustained more injuries to the head, face, and chest and were more frequently polytraumatized compared to conventional bicycle accidents.
2. A larger proportion of patients in the EB group sustained injuries with an AIS ≥ 2 in multiple body regions and received primary ICU treatment more frequently than those in the CB group.
3. Mortality rates are seven times higher in the octogenerians compared with younger patients upon e-bike accidents.
4. Younger patients were significantly more often involved in night-time accidents while under the influence of alcohol.

Despite the clinical relevance of e-bike accidents, as illustrated in the present study given their increasing incidence, literature on injury distribution and severity in large patient cohorts remains limited [4, 6, 11–13]. It was observed that patients in the EB group were significantly older than those in the CB group, which is in line with findings from, among others, Spörri et al. [14, 15]. Increased age itself is associated with a prolonged reaction time, decreased balance, and reduced postural stability which, together with the overall higher propulsion speeds achieved by e-bikes, most likely contributes to the increased prevalence of head, face, and chest injuries in the EB group with matching increased injury severity, contrary to the higher prevalence of spine, pelvic, and extremity injuries in the CB group [16–18].

Apart from having a higher rate of injury to multiple body parts, patients in the EB group were also significantly more likely to be polytraumatized, which explains our finding that patients were transported to the hospital significantly more often via air ambulance. These findings underpin the importance of using adequate personal protective equipment while e-biking as well as the possible necessity for optimizing legislation. Unfortunately, up until 2025, the TR-DGU did not record whether a helmet was worn by the cyclist, but several studies have shown that helmet use is associated with improved clinical outcomes such as higher scores for the Glasgow Coma Scale and fewer ventilator days in the ICU [19–21]. The increased number of polytrauma patients in the EB group was also consistent with the observation that primary ICU treatment was more frequently provided to patients in this group.

Furthermore, stratification of the EB cohort in different age groups identified a positive correlation between age and mortality, which is corroborated by findings from Rowh et al. [22]. Overall, our data show that in-hospital mortality of the EB group increased sevenfold across all age groups compared to the CB-group. Among patients aged 80 and over in the e-bike group, the mortality rate upon admission after an accident involving an e-bike was almost one-fifth. This indicates that the decision to ride an e-bike should be made with careful consideration of maximum speeds and the application of adequate personal protective equipment against the rider’s age. Furthermore, specific e-bike training could be considered as a preventive measure against e-bike accidents in the geriatric trauma population. Lastly, age stratification identified that roughly half of e-bike accidents in younger patients (age category 16–59) population occur under the influence of alcohol and/or at night, which is in line with results from Fernandez et al. [23]. These data indicate that further preventive measures as well as law enforcement may aid in preventing injuries.

The present study has limitations, first and foremost being the fact that the analyses can only be based on those variables that are documented in the TR-DGU. A further limitation is the loss to follow-up of patients transferred to a different hospital in the early post-traumatic phase. However, the number of excluded patients due to early transfers is low compared to the overall cohort. Lastly, although patients in the EB group were significantly more often polytraumatized, the older age can also be a confounding variable that contributed to the increased rate of ICU admission in this group.

## Conclusions

The present study is the first to report on injury patterns and mechanisms in e-bike accidents using the multicenter TR-DGU database. Our data show that patients in the EB group were significantly older, more frequently polytraumatized, and with significantly more injuries sustained to the head, face, and chest as compared to patients in the CB group. Moreover, it was demonstrated that in those patients involved in e-bike accidents, mortality rates increase significantly with rising age. Taken together, based on the obtained results, the present study underlines the need for personal protective equipment for all e-bike cyclists as well as carefull consideration of e-bike use for the elderly.

## Data Availability

The data presented in this study were made available from a third party, the AUC - Academy for Trauma Surgery (AUC - Akademie der Unfallchirurgie GmbH), but restrictions apply to the availability of these data, which were used under license for the current study, and are not publicly available. Data will be shared upon reasonable request and with permission of (AUC - Akademie der Unfallchirurgie GmbH, Emil-Riedel-Straße 5, 80538 München, Deutschland, Email: [support-tr@auc-online.de](mailto: support-tr@auc-online.de)).

## Abbreviations

AAAM: Association for the Advancement of Automotive Medicine
AIS: Abbreviated Injury Scale
AUC: Academy for Trauma Surgery (Akademie der Unfallchirurgie GmbH)
CB: Conventional Bicycle
CH: Switzerland (Confoederatio Helvetica)
D: Germany
DACH: Germany, Austria, Switzerland region
DGU: Deutsche Gesellschaft für Unfallchirurgie (German Trauma Society)
EB: E-bike
EMS: Emergency Medical Service (ground-based)
HEMS: Helicopter Emergency Medical Service
ICU: Intensive Care Unit
IQR: Interquartile Range
LOS: Length of Stay
SD: Standard Deviation
SPSS: Statistical Package for the Social Sciences
TBI: Traumatic Brain Injury
TR-DGU: TraumaRegister DGU^®^
WBCT: Whole-Body Computed Tomography
